# Effect of processing methods on nutritional, sensory, and physicochemical characteristics of biofortified bean flour

**DOI:** 10.1002/fsn3.301

**Published:** 2015-11-01

**Authors:** Marie Grace Nkundabombi, Dorothy Nakimbugwe, John H. Muyonga

**Affiliations:** ^1^ Department of Food Technology and NutritionMakerere UniversityKampalaUganda

**Keywords:** Extrusion cooking, iron biofortified beans, malting, mineral bioavailability, roasting

## Abstract

Common beans (*Phaseolus vulgaris* L.) are rich nutritious and affordable by vulnerable groups, thus a good choice for biofortification to address malnutrition. However, increasing micronutrients content of beans, without improving micronutrients bioavailability will not improve the micronutrients status of consumers. Effect of different processing methods on the physicochemical characteristics of biofortified bean flour was determined. Processing methods used in this study were malting (48 h), roasting (170°C/45 min), and extrusion cooking using a twin screw extruder with three heating sections, the first set at 60°C, the second at 130°C, and the last one at 150°C. The screw was set at a speed of 35 Hz (123g) and bean flour moisture content was 15%. Mineral extractability, in vitro protein digestibility, pasting properties, and sensory acceptability of porridge and sauce from processed flour were determined. All processing methods significantly increased (*P* < 0.05) mineral extractability, iron from 38.9% to 79.5% for K131 and from 40.7% to 83.4% for ROBA1, in vitro protein digestibility from 58.2% to 82% for ROBA1 and from 56.2% to 79% for K131. Pasting viscosities of both bean varieties reduced with processing. There was no significant difference (*P* < 0.05) between sensory acceptability of porridge or sauce from extruded biofortified bean flour and malted/roasted biofortified bean flour. Acceptability was also not affected by the bean variety used. Mineral bioavailability and in vitro protein digestibility increased more for extruded flour than for malted/roasted flours. Sauce and porridge prepared from processed biofortified bean flour had lower viscosity (extruded flour had the lowest viscosity), thus higher nutrient and energy density than those prepared from unprocessed biofortified bean flour. Estimated nutritional contribution of sauce and porridge made from processed ROBA1 flour to daily requirement of children below 5 years and women of reproductive age found to be high. These results show that processing methods enhanced nutritional value of biofortified bean flour and that processed biofortified bean flour can be used to prepare nutrient and energy‐dense gruel to improve on nutritional status of children under 5 years and women of reproductive age.

## Introduction

Micronutrient malnutrition especially iron deficiency anemia and zinc deficiency affect at least half of the world's population (Nestel et al. 2006). In Uganda, by considering iron deficiency anemia, the most affected age groups are children below 5 years (22%) and women of reproductive age (24%) (UBOS 2012). This is caused by low intake of daily required amount of micronutrients. Food fortification has been used as one of the approaches to overcome micronutrient deficiencies, but it is hard for vulnerable and poor people to access fortified processed food. Biofortification of popular food crops like beans was therefore proposed as a better intervention to address micronutrients malnutrition (Kimani et al. 2006; Pfeiffer and McClafferty 2007).

Common beans (*Phaseolus vulgaris* L.) are rich in protein (20–28%) and micronutrients (Fe = 34–89 mg/kg and Zn = 21–54 mg/kg) (Beebe et al. 2000). Beans are commonly consumed in East and central Africa (50–60 kg per capita) and are affordable by vulnerable groups making them a good choice for biofortification. Increasing micronutrients content of beans, without improving micronutrients bioavailability, will not have impact on micronutrients status of consumers (Petry et al. 2012). Bioavailability of micronutrients is limited by inhibitors, especially polyphenols and phytates (Gibson 1994; Luo and Xie 2012). Different processing techniques, such as germination, roasting, and extrusion cooking have been reported to reduce the level of antinutrients, thus to improve nutritive value of beans (Marzo et al. 2002; Audu and Aremu 2011). Processing can also improve cooking characteristics and physicochemical properties of beans. Extrusion processing reduces paste viscosity contributing to enhancement of nutrient content of dishes from beans, and making products suitable for vulnerable groups like children and women of child bearing age with high nutrient requirements (Edwards et al. 1994; Thaoge et al. 2003).

The aim of this study was to develop bean flour from biofortified beans, using malting, roasting, and extrusion processing, and evaluate the effect of these processing methods on the nutritional and physicochemical characteristics of the developed bean flour.

## Materials and Methods

### Preprocessing of bean flour

Iron biofortified bean variety (ROBA1) was purchased from Community Enterprise Development Organization (CEDO), a local nongovernmental organization (NGO) based in Rakai District. K131 beans were purchased from the Bean program of the National Crops Resource Research Institute (NaCRRI) in Namulonge, Uganda. K131 bean variety was selected because it is high yielding but takes long to cook due to a hard seed coat (Kalyebara 2005; Nyakuni 2008).

Two varieties of beans, ROBA1 and K131, were subjected to two different processing methods to produce flour. For each variety, 10 kg of beans were sorted. One part (5 kg) was soaked in distilled water at 1:2 (w/v) for 24 h. The soaked beans were germinated for 48 h in a dark place on a wet cloth. Germinated beans were then roasted at 170°C for 45 min in an oven (Infrared food oven GL‐2A, China) (Nakitto et al. 2015). After roasting, the beans were milled using a wonder mill (Grain of Truth Bread Company, Smithfield, North Carolina, USA). Flour was stored in plastic bags until further analysis. The second part (5 kg) of each bean variety were extruded using a twin screw extruder model DP‐70‐III (Jinan Eagle Food Machinery Co., Ltd. Jinan City‐Shandong Province, China) with three heating sections, the first was set at 60°C, the second at 130°C, and the last one at 150°C. The extruder filler was set at a speed of 30 Hz (900 rpm), the screw was set at a speed of 35 Hz (1050 rpm), and the cutters were set at a speed of 30 Hz (900 rpm). The diameter was 4 mm and the flour was extruded at 15% moisture content. The extruded beans were milled into flour and packed in plastic bags until further analysis.

### Formulation of composite flour

The composite flour was formulated using processed bean, amaranth, and rice flours. Amaranth and rice were previously ground into flour using a wonder mill (Bread of Truth Company). Amaranth, rice, and bean flours were mixed at a ratio of 30:30:40, respectively, a proportion reported to be highly acceptable (Ndagire et al. 2012).

### Determination of nutritional and physicochemical characteristics of the flours

#### Iron and zinc contents determination

Iron and zinc contents of raw, extruded, and malted/roasted bean flours of both K131 and ROBA1 bean varieties were analyzed by the method described by Duhan et al. (2002). One gram of sample was placed in 150 mL conical flash, and wet acid digested with 30 mL of nitric acid–perchloric acid mixture (HNO_3_:HClO_4_; 5:1 v/v) by heating until clear white precipitates settled at the bottom. The digested samples were dissolved in double distilled water and filtered through Whatman # 42 filter paper. The filtrate was made to 50 mL with double distilled water and used for determination of iron and zinc contents using Atomic Absorption Spectroscopy (AAS) (model; PerkinElmer 235, Norwalk, CT).

#### Iron and zinc extractability

Iron and zinc extractability were determined using a method by Duhan et al. (2002). The minerals in the samples were extracted with 0.03 N HCl by shaking in water bath (Grant OLS200, Cambridge, UK) at 37°C for 3 h. The clear extract obtained after filtration with Whatman # 42 filter paper was oven dried (M/S Scientific Instruments, New Delhi, India) at 100°C and wet acid digested. The amounts of the HCl‐extractable zinc and iron in the digested samples were determined by the methods described earlier for determination of mineral contents:

$$\text{Mineral extractability}\%=\frac{\text{Mineral extractable in 0.03 N HCl}}{\text{Total mineral}}\times 100$$

#### In vitro protein digestibility

##### Initial protein content

Protein content was determined by Kjeldahl method #46‐12 (AACC 2004). Approximately 0.5 g sample was weighed into digestion flasks into which 1 g of potassium sulfate, 1 mL of 10% copper sulfate, and 10 mL of concentrated sulfuric acid were added. A blank with no sample was similarly prepared. The flasks were heated on a digestion rack until white fumes were emitted, and the heating continued for another 2 h.

Flasks were cooled, after which the digests were transferred quantitatively to 50 mL volumetric flasks, made to volume with distilled water, and mixed immediately. Distillation of samples was done by pipetting 50 mL of sample into the distillation chamber and slowly adding approximately 50% NaOH solution to raise the pH. A conical flask containing 10 mL of 2% boric acid solution plus two drops of bromocresol green methyl red indicator was placed under the condenser stem to collect the distillate. The distillation was allowed to proceed for 7.5 min after which the receiving flasks were lowered so that the distillate could wash any remaining ammonia from the tip of the condensing unit. Titration was done using a burette filled with 0.05 N H_2_SO_4_. The end point was determined by the sample solution turning from blue‐green to pink. The volume of the acid used (titer) was noted. The percentage crude protein (% cP) in the sample was calculated using the following formula:

$$\%\text{cP}=\frac{T\times 14\times b\times 50\times 100\times 6.25}{1000\times 5\times \text{Wt}}$$

*T*: titer; 14: atomic weight of N; b: normality of acid which was approximate 0.05 N; Wt: sample weight; 6.25: conversion factor for protein from % nitrogen.

##### Pepsin digestion

This was determined using the method of Mertz et al. (1984). Approximately 0.2 g of the samples was weighed into 50 mL centrifuge tubes. To each sample, 2 mL of distilled water was added, shaken, and the tubes were then placed in a boiling water bath for 20 min. Phosphate buffer with pepsin solution was added to the mixtures (20 mL of 0.1 mol/L phosphate buffer and 1:3000 IU Hog pepsin/L, pH 2.0). A blank was prepared in a similar way without the sample. The tubes were incubated in a shaking water bath (Grant OLS200, Cambridge, UK) at 37°C for 2 h and then centrifuged at 4032 g (225, Fisher Scientific, Missouri City, Texas, USA) for 15 min and the supernatant was removed with a dropper and discarded. To each tube, 10 mL of phosphate buffer was added and centrifuged at 4032 g and the supernatant was discarded. The residue was removed and placed in the center of a filter paper on a Buchner funnel. Suction was applied to the filter flask and the remaining residue was rinsed from the tube into the funnel using 5 mL buffer. The filter paper were rolled and inserted into Kjeldahl flasks containing filter paper and sample, and concentrated H_2_SO_4_ (10 mL), 1 g of potassium sulfate, and 1 mL of 10% copper sulfate solution were added. Digestion, distillation, and titration were done to determine the protein content.

$$\text{In vitro protein digestibility}\;=\frac{A-B}{A}$$

where *A* is % protein in sample before digestion and *B* is % protein in sample after pepsin digestion

#### Determination of pasting properties

Pasting properties were determined using the Rapid Visco Analyzer (RVA‐4, Newport Scientific Pty Ltd., Warriewood, NSW, Australia) with Thermocline for windows software (AACC # 76‐21, 1999). Four grams of flour was suspended in 25 mL of distilled water in an RVA canister. The canister was loaded into the RVA and analyzed with a constant speed (3 g). The holding viscosity, peak viscosity, final viscosity, and pasting temperature of bean flour and bean‐based composite flour were determined in duplicates.

### Consumer acceptability of porridges and sauces prepared using the processed flour of biofortified and conventional beans

#### Porridge preparation

Porridge was prepared from both extruded and malted/roasted bean flours of both the ROBA1 and K131 bean varieties. The flour (250 g) was mixed with 750 mL of cold water. The mixture was brought to the boil while stirring to avoid lumping then cooked for 10 min after which 50 g of sugar was added.

#### Sauce preparation

Sauce was prepared from both extruded and malted bean flours of both the ROBA1 and K131 bean varieties. Ingredients (26 g of onions or ½ of an onion; 23 g or ½ a green pepper; 69 g or 1 medium sized tomato) were all chopped. The onions and green pepper were shallow fried in 50 mL of vegetable cooking oil. About 2 g of curry powder and 10 g of salt were added. The bean flour (250 g) was stirred into the mixture, 1200 mL of water was added gradually with constant stirring to avoid lumping, and the sauce was cooked for 10 min.

Porridge and sauce acceptability were determined by 50 panelists using a 9‐point hedonic scale (Kemp et al. 2009). The flavor, color, appearance, thickness, texture, taste, smell, and overall acceptability were determined.

### Nutrients and energy density of flour from biofortified beans

The processed flour rate, which resulted in porridge or sauce with a spoonable consistency (2500–3000 cP) (Thaoge et al. 2003), was determined by measuring viscosities of the sauce and porridge using Brookfield Viscometer (Model DVII Rheometer V2.0 RV; Middleboro, MA). The nutrient content of the gruels with spoonable viscosities were computed and compared to the recommended daily intake of iron, zinc, protein, and energy (Table 1) for children below 5 years and women of reproductive age (15–49 years). The comparison was based on flour rate and nutrient and energy density of each sauce and porridge prepared from unprocessed flour, malted/roasted bean flour, and extruded bean flour. For children aged between 1 and 5 years, contribution of sauce/porridge to their energy and nutrients intake was computed by considering 300 mL of sauce/porridge as the serving (Pipes and Trahms 1994; Thaoge et al. 2003), whereas for women of reproductive age (15–49 years), 500 mL of sauce/porridge was considered as the serving. The contribution of iron and zinc to dietary requirements was calculated based on bioavailable content, while for protein it was calculated based on digestible protein content.

**Table 1:** Recommended daily intake of iron, zinc, protein, and energy for target groups

Age/sex	Iron (mg/day)	Zinc (mg/day)		Protein (g/day)	Energy (kcal/day)
Male					
1–3 years	7	3		13	1046
4–5 years	10	5		19	1742
Female					
1–3 years	7	3		13	992
4–5 years	10	5		19	1642
15–18 years	15	9		46	2368
19–49 years	18	8		46	2403
Pregnancy					
≤18 years		12	First trimester	46	
19–49 years	27	11	Second trimester	71	(+340)
			Third trimester	71	(+452)
Lactation					
≤18 years	10	13	First 6 months	71	(+330)
19–49 years	9	12	Second 6 months	71	(+400)

Source: Adapted from the Dietary Reference Intake series, National Academies Press. Copyright 1997, 1998, 2000, 2001, 2002, 2004, 2005 by the National Academy of Sciences, (Rolfes et al. 2011).

### Data analysis

Data were entered into excel spread sheet and subjected to analysis of variance (ANOVA) using Statistix software (version 9.0) at *P* ≤ 0.05.

## Results and Discussion

### Effect of different processing methods on the nutritional and physicochemical characteristics of iron biofortified bean flour

#### Iron and zinc contents of flours of ROBA1 and K131 bean varieties

The iron and zinc contents of ROBA1 beans were higher than that of K131 (Table 2). ROBA1 is a micronutrient‐enriched variety and is one of the 960 bean population that have been developed and identified by the national research programs of east and central African countries, as being high in iron (above 70 mg/kg compared to 50 mg/kg for conventional beans) and zinc (above 30 mg/kg compared to 20 mg/kg for conventional beans) (Ugen et al. 2012).

**Table 2:** Results for iron and zinc contents of flours from ROBA1 and K131 bean varieties

Sample	Raw (mg/kg)	Malted/roasted (mg/kg)	Extruded (mg/kg)
Iron			
ROBA1	70.25 ± 1.5^a^	58.40 ± 0.6^a^	83.50 ± 0.56^a^
K131	66.45 ± 1.5^b^	47.75 ± 2.6^b^	75.85 ± 1.03^b^
Mean	68.35^a^	53.07^b^	79.67^c^
Zinc			
ROBA1	26.75 ± 0.2^a^	23.00 ± 1.00^a^	22.65 ± 0.73^a^
K131	23.00 ± 0.7^b^	22.95 ± 0.04^a^	21.25 ± 0.75^a^
Mean	24.87^a^	22.97^b^	21.95^b^

Means in each column with different superscripts are significantly different (*P* < 0.05).

The last row of each mineral compares processing methods across the row (*P* < 0.05).

The results of iron and zinc contents obtained were in the same range as that reported by Blair et al. (2009) between 40.0 and 84.6 mg/kg for iron and 17.7 and 42.4 mg/kg for zinc. The recorded iron and zinc contents for unprocessed sample were higher than the respective values of 51.1 and 24.9 mg/kg reported by Tryphone and Nchimbi‐Msolla (2010), except the zinc content of K131. The data for iron and zinc contents of ROBA1 are higher than those reported for ROBA1 beans grown in Ethiopia (63.13 and 15.9 mg/kg, respectively) (Shimelis and Rakshit 2005). This difference may be due to the different growing field conditions.

Processing by germination/malting followed by roasting resulted in a slight and statistically significant decrease in mineral content of both the ROBA1 and K131 bean varieties compared to the mineral content of raw flour. This can be attributed to leaching of iron and zinc ions into the soaking water (Afify et al. 2011; Carvalho et al. 2012). After extrusion, the iron content of both varieties apparently increased (about 58% for ROBA1 and 50% for K131). A similar observation was made in other studies (Alonso et al. 2001; Camire 2002; Murekatete et al. 2010; Mutambuka 2013); according to Camire (2002), this increase may be attributed to the migration of iron from extruder parts, manly screws. After extrusion, zinc content of both samples decreased. Murekatete et al. (2010) also reported significant changes in mineral content after extrusion cooking where iron content increased and zinc content decreased. The reported increase in iron content was 50% for one sample and 30% for the second sample; for Camire (2002), the increase was 38% in iron after extrusion cooking, while for Alonso et al. (2001), the increase in iron after extrusion was 20% for pea and 76% for kidney beans.

There were significant differences in both zinc and iron contents (*P* < 0.05) of the ROBA1 and K131 bean varieties studied in this work. Processing malting/roasting and extrusion also significantly (*P* < 0.05) affected the iron and zinc contents of flour compared to raw beans flour.

#### Iron and zinc extractability

The iron extractability was in a range of 38.92–83.41%, while for zinc the range was between 51.5% and 73.68% (Table 3).

**Table 3:** Results for iron and zinc extractability of flours from ROBA1 and K131 bean varieties (%)

Sample	Raw	Malted/roasted	Extrusion
Iron			
ROBA1	40.75 ± 1.06^d^	74 ± 1.41^c^	83.41 ± 1.34^a^
K131	38.92 ± 1.02^d^	70.5 ± 2.12^c^	79.45 ± 0.91^b^
Mean	39.83^c^	72.25^b^	81.43^a^
Zinc			
ROBA1	51.5 ± 2.12^c^	68.62 ± 0.8^b^	73.68 ± 1.8^a^
K131	55 ± 1.41^c^	66.89 ± 1.2^b^	72.28 ± 2.4^a^
Mean	53.25^c^	67.75^b^	72.98^a^

Means in each column with different superscripts are significantly different (*P* < 0.05).

The last row of each mineral compares processing methods across the row (*P* < 0.05).

A significant difference (*P* < 0.05) was observed between the mineral extractability of processed bean flour samples and unprocessed bean flour samples for both the ROBA1 and K131 bean varieties. Flour of extruded beans had the highest iron and zinc extractabilities. Similar results were reported for the effect of processing on mineral bioavailability (Khatoon and Prakash 2006; Viadel et al. 2006; Al‐numair et al. 2009). On comparing varieties, a significant difference in iron extractability is observed with ROBA1 having higher extractability than K131. Results for zinc extractability, however, did not reveal any significant differences between the two bean varieties.

Increase in mineral extractability following the malting/roasting process can be attributed to reduction in phytate and polyphenol contents which are inhibitors of mineral absorption. Increase in enzymatic activity during malting (phyatase and polyphenoloxidase) results in enzymatic degradation of phytates and polyphenols (Savelkoul et al. 1992; Reddy and Pierson 1994; Alonso et al. 1998; Sandberg, 2002). Soaking, which is part of the malting process, was also reported to reduce phytates by water solubilization and subsequent leaching of some phytic acid salts (Afify et al. 2011). Roasting has also been reported to reduce antinutrients, especially phytates, by increasing phytase (El‐adaway 2002; Ramakrishna et al. 2006; Afify et al. 2011; Subuola et al. 2012). In addition, extrusion cooking was also reported to improve mineral bioavailability by reducing other factors that inhibit absorption such as phytates. The high extrusion temperatures were proposed to result in phytate hydrolysis, resulting in higher availability of minerals after processing than other processing methods (Alonso et al. 2001; Singh et al. 2007).

#### In vitro protein digestibility

Of the two bean varieties used in this study, K131 had higher protein content (Table 4). However, there was no significant difference between the protein content of raw and extruded bean flours of both varieties. Malted/roasted samples, however, had higher protein content compared to raw and extruded samples. Osman (2007) reported the similar increase in protein content after malting/germination, he related this result to increasing water activity during germination due to hydrolytic enzymes.

**Table 4:** Protein content (g/100 g) of the raw, extruded, and malted/roasted ROBA1 and K131 bean flours

Bean variety		Protein content (g/100 g)	Digestibility (%)
ROBA1	Raw	20.65 ± 0.8^c^	58.27 ± 1.5^c^
	Extruded	20.97 ± 0.1^c^	82.00 ± 1.4^a^
	Malted	21.37 ± 0.2^c^	72.50 ± 1.7^b^
K131	Raw	23.14 ± 0.04^b^	56.28 ± 0.04^c^
	Extruded	21.69 ± 0.3^c^	79.00 ± 1.6^a^
	Malted/roasted	24.66 ± 0.9^a^	70.50 ± 0.7^b^

Means in each column with different superscripts are significantly different (*P* < 0.05).

Increase in protein content in malted beans has been reported in other studies and attributed to mobilization of protein reserves in cotyledons which take place during malting, together with the synthesis of new proteins necessary for growth of the sprout (El‐adaway 2002; Rodriguez et al. 2008; Taraseviciene et al. 2009). Wang et al. (2008) attributed this increase in protein content to the loss of soluble solids in soaking water done before malting. Protein content of ROBA1 variety in this study was quite similar to the protein content reported by Shimelis and Rakshit (2005) for ROBA1 variety grown in Ethiopia (20.5 g/100 g).

In vitro protein digestibility increased significantly (*P* < 0.05) after both malting/roasting and extrusion cooking, and for both varieties compared to raw samples. Extruded samples had a significantly higher in vitro protein digestibility than samples processed by malting and roasting. There was also a significant difference (*P* < 0.05) between the in vitro protein digestibility of the two bean varieties with ROBA1 bean flour having a higher in vitro protein digestibility. Similar results were reported by Shimelis and Rakshit (2007) who observed a higher in vitro protein digestibility of ROBA1 variety compared to two other varieties studied. However, the in vitro protein digestibility values reported by their study were generally higher than those in the current study. Improvement of in vitro protein digestibility after processing may be not only due to removal or reduction of antinutrients, but may also be attributed to breakdown of the native protein structure, including enzyme inhibitors and lectins; differential solubility of oligosaccharides and their diffusion rates; phytase activity to break down phytic acid in the seeds; and the development of endogenous *α*‐galactosidase activity to reduce oligosaccharides. Combination of processing methods has been reported to be more effective than use of single treatments, especially when one of the methods is heat processing (Shimelis and Rakshit 2007). Extrusion has been reported to improve protein digestibility by reducing antinutrient factors (Prakrati et al. 1999).

#### Pasting properties

**Figure 1:**
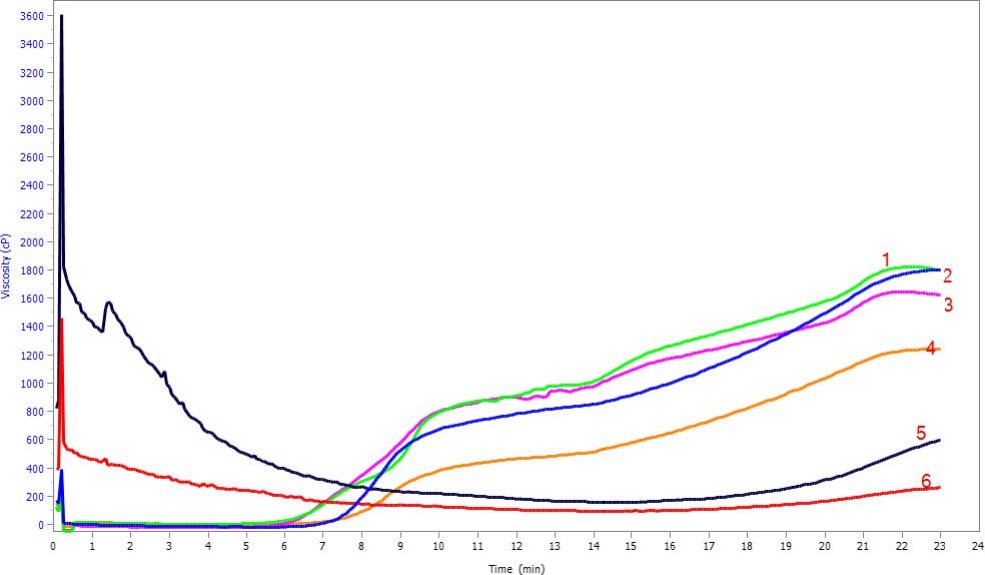
Pasting properties of unprocessed and processed ROBA1 and K131 bean flours.

Significant differences (*P* < 0.05) were observed in the different pasting characteristics of flours from both processing methods and varieties (Table 5; Fig. 1). There were no significant differences between the two varieties studied for pasting temperature, setback viscosity, trough viscosity, and peak viscosity. Pasting temperature of flours ranged from 94.9°C to 53.2°C, being significantly lower (*P* < 0.05) for extruded flour compared to raw (unprocessed) and malted/roasted flours. Pasting temperature of raw flour was higher than reported by Akinjayeju and Ajayi (2011) for black bean flour (80–82°C). Extruded flour had the lowest pasting temperature (53.2°C for K131 flour and 54.7°C for ROBA1 flour). Jozinovic et al. (2012) reported a decrease in pasting temperature after extrusion of corn meal. Pasting temperature is an indication of the minimum temperature required to cook the flour (Kaur et al., 2007). Previous studies reported that germination/malting did not have any effect on pasting temperature of flours (Moongngarm 2011; Ritruengdech et al. 2011; Borijindakul and Phimolsiripol 2013), and Sade (2009) in his study on pearl millet (*Pennisetum glaucum*) did not observe any effect on pasting temperature due to roasting or germination of pearl millet. Similarly, malting/roasting of beans in this study had no effect on pasting temperature. High pasting temperature may be an indication of the presence of resistant starch, to swelling and rupturing (Kaur and Singh, 2007).

**Table 5:** Pasting properties of raw, extruded, and malted/roasted ROBA1 and K131 bean flours

Sample		PT (°C)	PV (cP)	TV (cP)	BV (cP)	FV (cP)	SV (cP)
ROBA1	Raw	94.9 ± 1.6^a^	906 ± 2.7^ab^	871.6 ± 3.9^ab^	6 ± 1.1^c^	1539 ± 2.2^b^	667.3 ± 2.5^c^
	Extruded	54.7 ± 3.1^c^	488.3 ± 2.1^c^	156.33 ± 0.7^d^	749.6 ± 2.7^a^	631 ± 0.96^d^	474.7 ± 0.8^d^
	Malted/roasted	93.9 ± 0.2^ab^	877.6 ± 0.7^ab^	490.00 ± 2.2^c^	−1.6 ± 1.15^c^	1264.7 ± 1.3^c^	774.7 ± 2.3^bc^
K131	Raw	94.9 ± 0.02^a^	979 ± 2.9^a^	980 ± 4.5^a^	−1 ± 1.7^c^	1810.7 ± 1.8^a^	830.7 ± 1.9^b^
	Extruded	53.2 ± 0.49^c^	399.6 ± 2.1^c^	129 ± 1.6^d^	270.6 ± 2.3^b^	387.3 ± 1.9^e^	258.3 ± 2.4^e^
	Malted/roasted	90.3 ± 3.2^b^	798.3 ± 3.0^b^	798.6 ± 2.3^b^	−0.3 ± 1.7^c^	1799 ± 1.4^a^	1000.3 ± 1.5^a^

PT, pasting temperature; PV, peak viscosity; TV, trough viscosity; BV, breakdown viscosity; FV, final viscosity; SV, setback viscosity.

Means in each column with different superscripts are significantly different (*P* < 0.05).

The highest peak viscosity was observed for raw flour (906 cP for ROBA1 flour and 979 cP for K131 flour), while the lowest was observed for extruded flour (399.6 cP for K131 flour and 488.3 cP for ROBA1 flour). Peak viscosity is the maximum viscosity attained by gelatinized starch during heating in water. It indicates the water binding capacity of the starch granules (Shimelis et al., 2006). In the present study, processing methods were observed to reduce peak viscosity. The low peak viscosity values of extruded compared to unprocessed flours may be attributed to the denaturation of protein as well as the starch–protein interactions which result in structures with low capacity for interaction with water (Hernandez‐Nava et al. 2011). The highest trough viscosity value was recorded for raw K131 flour (980 cP) and the lowest was recorded for extruded K131 flour (129 cP). Breakdown viscosity (measure of the ease with which the swollen granules can be disintegrated) (Kaur and Singh, 2007) ranged from −1.67 cP for malted/roasted ROBA1 flour to 749 cP for extruded ROBA1 sample. Extruded flour exhibited significantly (*P* < 0.05) higher breakdown viscosity (270.6 cP for extruded K131 flour and 749.6 cP for extruded ROBA1 flour) than the malted and unprocessed flours. Pastes from flours with low final viscosity were reported to be less stable when cooked and thus to commonly have high values of breakdown viscosity (Ikegwu et al. 2010). Similarly, in the current work, extruded flour had low final and high breakdown viscosities. ROBA1 beans exhibited higher breakdown viscosity values compared to K131, regardless of processing method. High breakdown viscosity is indicative of lower ability of starch to resist shear stress during cooking (Adebowale et al. 2005). The results of this study therefore indicate that extended cooking under shear would lead to significant alterations in extruded samples compared to malted or raw (unprocessed) samples.

Final viscosity (which indicates the ability of the starch to form a viscous paste (Ashogbon and Akintayo 2012) ranged from 387 cP to 1, 810.7 cP, with no significant difference between raw (unprocessed) flour and malted/roasted flour. However, the final viscosity for extruded samples was significantly lower (387.3 cP for extruded K131 flour and 631 cP for extruded ROAB1 flour) than the raw (unprocessed) and malted flour. Of the two varieties studied, ROBA1 exhibited lower final viscosity compared to K131. Generally, the final viscosities of all samples were higher than other viscosity values in the pasting cycle. This is attributable to the reassociation of amylose molecules (Miles et al. 1985; Ashogbon and Akintayo 2012). Setback viscosity (measure of retrogradation tendency of flours upon cooling of cooked flour pastes; Kaur and Singh, 2007) ranged from 258 cP for extruded K131 to 1000.3 cP for malted/roasted K131. Extruded samples exhibited the lowest setback viscosity (258.3 cP for extruded K131 flour and 474 cP for extruded ROBA1 flour). This implies that extrusion cooking reduced the retrogradation tendency of flour. Gonzalez and Perez (2002) also reported a reduction in setback viscosity after extrusion cooking of lentil starches. This may be due to starch degradation during extrusion cooking (Ozcan and Jackson 2005).

Borijindakul and Phimolsiripol (2013) reported that germination reduced all pasting viscosities, as observed in the present study. During germination/malting, there is an activation of *α* amylase which hydrolyzes starch thus reducing viscosity (Moongngarm 2011; Ritruengdech et al. 2011).

During extrusion cooking, starch is pregelatinized and when the pregelatinized starch granules are heated with water, swelling, rupture, crystallinity loss, and amylose leaching occur. Extruded flour could thus absorb water and become viscous instantly, but it recorded low paste viscosity (Ritruengdech et al. 2011). Hagenimana et al. (2006) also reported a decrease in all viscosity values of extruded rice flour compared to unprocessed rice flour, like this study. Extrusion cooking has been used to reduce viscosity in other to increase energy density of gruels (Onyango et al. 2004; Magala‐nyago et al. 2005).

Processing methods used in the present study generally reduced pasting viscosity. This leads to increase in the flour rate or solid matter when preparing porridge to reach the acceptable viscosity (2500–3000 cP) (Thaoge et al. 2003), resulting in porridges with higher nutrient and energy density.

### Consumer acceptability of porridges and sauces prepared using flours of extruded or malted biofortified or conventional beans

#### Acceptability of porridges from both bean varieties

The overall acceptability was highest for porridges prepared from the composite flour of both extruded and malted/roasted ROBA1 beans which scored 7 on the 9‐point hedonic scale (Table 6). The composite porridges were significantly more acceptable (*P* < 0.05) than those prepared from pure malted/roasted ROBA1 and K131 flours. There was no significant difference in the acceptability of composite porridges prepared from K131 and extruded pure ROBA1 (*P* < 0.05). The color of composite malted/roasted K131 porridge and extruded pure K131 porridge were significantly different and less acceptable than the colors of the other porridges. Moreover, for thickness and texture of extruded pure K131 and malted/roasted pure K131 porridges were also significantly different from other porridges (*P* < 0.05). Similarly, the appearances of the extruded composite K131and extruded pure K131 porridges were less acceptable than the other porridges (*P* < 0.05). For other attributes no significant differences were observed.

**Table 6:** Acceptability of porridge prepared from processed ROBA1 and K131 bean flours

	Samples	Taste	Flavor	Color	Smell	Thickness	Texture	Appearance	Overall acceptability
Malted/roasted	Pure ROBA1	5.43 ± 2.1^a^	5.25 ± 2.0^a^	6.55 ± 1.8^a^	5.48 ± 2.1^a^	5.90 ± 1.8^a^	5.82 ± 1.79^a^	6.33 ± 2.1^ab^	6.22 ± 1.5^b^
	Pure K131	6.23 ± 2.2^a^	6.20 ± 2.0^a^	6.75 ± 1.7^a^	6.18 ± 1.8^a^	6.65 ± 1.6^a^	6.30 ± 1.41^a^	6.83 ± 1.4^a^	6.07 ± 1.2^b^
	Composite ROBA1	6.55 ± 1.5^a^	6.07 ± 1.7^a^	6.15 ± 1.6^ab^	6.15 ± 1.6^a^	6.40 ± 1.5^a^	6.45 ± 1.26^a^	6.18 ± 1.6^ab^	7 ± 0.9^a^
	Composite K131	6.07 ± 1.8^a^	6.18 ± 1.6^a^	5.33 ± 2.0^b^	5.92 ± 1.4^a^	6.00 ± 1.7^a^	6.00 ± 1.72^a^	5.30 ± 1.8^b^	6.52 ± 1.4^ab^
Extruded	Pure ROBA1	6.85 ± 1.6^a^	5.9 ± 1.6^a^	5.68 ± 1.8^ab^	5.92 ± 2.0^a^	5.55 ± 2.3^a^	6.35 ± 1.9^a^	7.30 ± 1.7^a^	6.4 ± 1.6^ab^
	Pure K131	5.70 ± 1.9^a^	5.65 ± 1.7^a^	4.75 ± 1.7^b^	4.98 ± 2.1^a^	4.72 ± 2.6^b^	4.72 ± 2.6^b^	6.15 ± 2.4^b^	5.78 ± 1.6^b^
	Composite ROBA1	6.87 ± 1.9^a^	6.07 ± 1.7^a^	6.10 ± 1.8^a^	6.05 ± 1.9^a^	6.85 ± 2.0^a^	6.98 ± 1.5^a^	7.73 ± 1.9^a^	7 ± 1.7^a^
	CompositeK131	6.00 ± 1.8^a^	5.85 ± 1.6^a^	5.12 ± 1.7^ab^	5.70 ± 1.6^a^	6.28 ± 2.3^a^	6.55 ± 2.0^a^	5.80 ± 1.8^b^	6.18 ± 1.4^ab^

Means in each column with different superscripts are significantly different (*P* < 0.05).

#### Acceptability of sauces of bean varieties

Sauces prepared from extruded and malted/roasted pure ROBA1 and K131 flours had higher overall acceptability than the composite sauces (Table 7). The taste of the sauce made from malted/roasted composite flour was significantly different from other sauces (*P* < 0.05). The flavor of sauces made from extruded pure K131 and extruded composite ROBA1 was significantly less liked than the flavor of the other sauces (*P* < 0.05). The color, thickness, and appearance of sauces made from extruded pure K131 flour, malted/roasted composite ROBA1, and malted/roasted composite K131 flour were less accepted than for other sauces (*P* < 0.05). No difference was found for smell among all sauces.

**Table 7:** Acceptability of sauce made from processed ROBA1 and K131 bean flours

	Samples	Taste	Flavor	Color	Smell	Thickness	Texture	Appearance	Overall acceptability
Extruded	Pure ROBA1	7.0 ± 1.6^a^	6.65 ± 1.3^a^	6.55 ± 1.8^a^	6.17 ± 2.1^a^	6.20 ± 1.9^a^	6.60 ± 1.4^a^	6.50 ± 1.7^a^	7.0 ± 1.^a^
	Pure K131	6.0 ± 2.1^a^	5.55 ± 1.9^b^	5 ± 1.7^b^	5.35 ± 2.2^a^	4.80 ± 2.5^b^	5.23 ± 1.8^b^	5.08 ± 1.7^b^	6.12 ± 1.5^b^
	Composite ROBA1	6.0 ± 2.3^a^	6.0 ± 1.5^b^	6.45 ± 1.7^a^	5.40 ± 2.2^a^	6.52 ± 1.9^a^	6.70 ± 1.6^a^	6.70 ± 1.6^a^	6.63 ± 1.7^ab^
	Composite K131	6.4 ± 1.9^a^	6.18 ± 1.3^ab^	5.85 ± 1.6^a^	5.87 ± 1.6^a^	6.92 ± 1.7^a^	7.00 ± 1.9^a^	6.17 ± 1.4^a^	6.22 ± 1.9^ab^
Malted/roasted	Pure ROBA1	5.92 ± 2.05^ab^	5.72 ± 1.83^a^	7.55 ± 2.01^a^	6.0 ± 1.88^a^	7.07 ± 1.91^a^	5.97 ± 2.1^a^	7.4 ± 2.11^a^	7.0 ± 1.63^a^
	Pure K131	6.70 ± 2.03^a^	6.30 ± 1.87^a^	6.63 ± 1.1^a^	6.3 ± 2.18^a^	7.1 ± 1.78^a^	6.3 ± 1.9^a^	7.20 ± 1.3^a^	7.0 ± 1.50^a^
	Composite ROBA1	5.48 ± 2.19^b^	5.52 ± 2.20^a^	5.20 ± 1.9^b^	6.05 ± 1.78^a^	5.32 ± 2.1^b^	5.58 ± 2.1^a^	5.2 ± 2.21^b^	6.0 ± 1.65^b^
	Composite K131	5.92 ± 1.93^ab^	5.55 ± 1.82^a^	5.18 ± 1.8^b^	6.10 ± 1.82^a^	5.8 ± 1.28^b^	5.20 ± 1.2^a^	4.7 ± 1.45^b^	5.45 ± 1.23^b^

Means in each column with different superscripts are significantly different (*P* < 0.05).

The composite flour of ROBA1 made the most acceptable porridge after both processing methods (extrusion and malting/roasting). Similarly, the sauce made from the pure ROBA1 flour was the most acceptable after extrusion processing, while for malting/roasting method, both K131 and ROBA1 pure sauce were equally acceptable. Sensory scores of all products were higher than 5, with overall acceptability scoring higher than 6 (which correspond to 6 = like slightly, 5 = neither like nor dislike). The high acceptability may be attributed to the elimination of the beany flavor, which characterizes all legumes. Products prepared from beans are likely to have this flavor which is considered unpleasant to most consumers (Enwere 1998). Processes such as roasting and extrusion cooking are reported to decrease this beany flavor thus improve sensory attributes of bean based products (Nyombaire et al. 2011).

Overall, when the acceptability of the sauces and porridges is compared, porridge from the composite flour was more acceptable than that from the pure bean flour while sauce from the pure bean flour was more acceptable than that from the composites. Jackson et al. (2013) also reported that composite sorghum–bean porridge was more accepted than sorghum porridge. It is therefore recommended that the pure flour be promoted for use as sauces and the composite flour be promoted for use as porridges.

### Nutrients and energy density of flour from biofortified beans

Raw (unprocessed), malted/roasted, and extruded beans flour from ROBA1 were used to determine the flour rate which resulted in spoonable consistency (2500–3000 cP) (Thaoge et al. 2003) (Table 8). Unprocessed flour required a lower flour rate compared with processed flour, and extruded flour required the highest flour rate. Current results are in the same range with what was reported by Rombo et al. (2001) in a study irradiation processing for maize and kidney bean flour. Most of traditional porridges or gruels are made from cereal‐based food, and their flour concentration is between 5% and 10% to reach the maximum consistency (3000 cP) (Lorri 1993; Wambugu et al. 2003), the current flour had higher flour concentration.

**Table 8:** Flour concentrations of unprocessed and processed ROBA1 bean flours

Samples	Flour rate (%)	Viscosity (cP)
Unprocessed bean flour	10	1530
	13	2400
	**15**	**2859** a
	17	3229
Extruded flour	15	1700
	20	2571
	**22**	**2815** a
	23	3009
Malted/roasted flour	15	1684
	20	2603
	**21**	**2770** a
	22	3100

a Selected to compare nutrient density.

**Figure 2:**
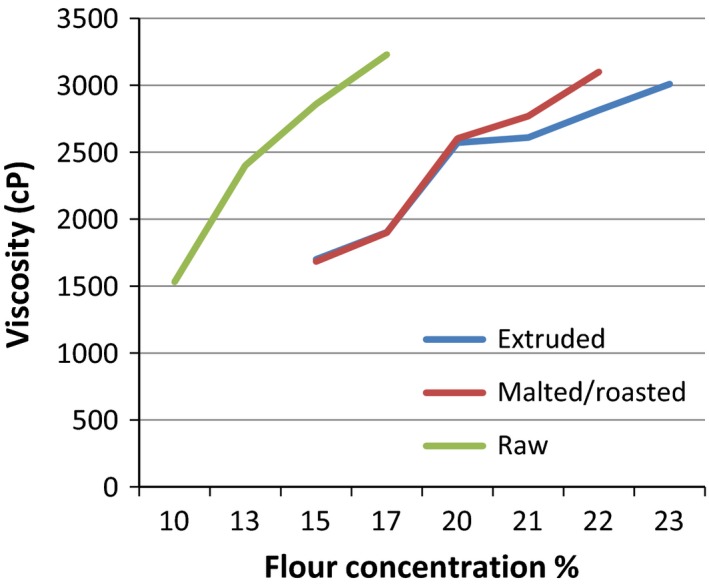
Variation in the viscosity of unprocessed and processed bean pastes with flour concentration.

Unprocessed flour (raw) attained the maximum viscosity (3000 cP) at a lower flour concentration compared to extruded and malted/roasted flours (Fig. 2 and Table 8). Malted/roasted flour attained the viscosity range at a slight lower flour concentration compared to extruded flour, but the difference was not significant.

Energy and nutrient density of both sauce and porridge were computed based on their flour rates. The highest flour rates within the range were selected for comparison (Table 9). Sauce prepared from unprocessed flour had significantly lower nutrients and energy density compared to malted/roasted flour and extruded flour. Extruded sauce had a slightly higher nutrients and energy density than malted/roasted sauce, but the difference was not significant. This difference may be due to the high nutrient bioavailability of extruded ROBA1 flour and also the high flour concentration. Similar results were reported by Saalia et al. (2012) who reported that porridge or gruel prepared with low solid content had low nutrients and energy density. Consequently, consumers especially children need to take a large volume of such porridge to meet their daily requirement. There is a risk that children do not meet their nutrient requirements given their limited stomach capacities.

**Table 9:** Energy and nutrients densities of sauces (100 mL) prepared from malted and extruded bean flours

Parameters		Unprocessed flour (15%)	Malted/roasted flour (21%)	Extruded flour (22%)
Energy (Kcal)		49.95	90.72	99.12
Iron	Total content (mg)	1.05	1.23	1.8
	Extractable (mg)	0.43	0.91	1.50
Zinc	Total content (mg)	0.40	0.48	0.51
	Extractable (mg)	0.21	0.33	0.37
Protein	Total content (g)	3.09	4.50	4.77
	Digestible (g)	1.80	3.26	3.91

#### Contribution of sauces to the recommended dietary intake of key nutrients and energy intake for vulnerable groups

Women of reproductive age and young children are the most affected by malnutrition, especially micronutrient deficiency (iron and zinc). The contribution of sauces to the recommended dietary intake (Table 10) was calculated and compared. It was observed that extruded sauce supplied the highest amount of nutrients (iron, zinc, and protein) and energy followed by malted/roasted sauce. Sauce from unprocessed flour supplied the lowest amounts of nutrients. The estimates were based on three servings per day (300 mL of sauce/porridge per serving for children and 500 mL of sauce/porridge per serving for women of reproductive age).

**Table 10:** Contribution (%) of three servings per day of sauces to the recommended daily intake of children and women of reproductive age

Age/sex	Iron (%)			Zinc (%)				Protein (%)			Energy (%)		
	US	MS	ES	US	MS	ES		US	MS	ES	US	MS	ES
Male													
1–3 years	55	117	193	51	100	112		125	226	270	43	78	85
4–5 years	39	82	135	37	60	67		85	154	185	26	47	51
Female													
1–3 years	55	117	193	51	100	112		125	226	270	45	82	90
4–5 years	39	82	135	37	60	67		85	154	185	27	50	54
15–18 years	43	91	150	34	55	62		59	106	127	32	57	63
19–49 years	34	76	126	39	62	70		59	106	127	31	57	62
Pregnancy													
≤18 years				26	41	46	First trimester	59	106	127	31	57	62
19–49 years	24	50	83	28	45	51	Second trimester	38	69	82	27	50	54
							Third trimester	38	69	82	26	48	52
Lactation													
≤18 years	64	136	225	24	38	43	First 6 months	38	69	82	27	50	54
19–49 years	71	151	250	26	41	46	Second 6 months	38	69	82	27	48	53

US, sauce from unprocessed flour; MS, sauce from malted/roasted flour; ES, sauce from extruded flour.

For children (1–5 years), the lowest nutritional contribution for iron, zinc, protein, and energy were 38.6%, 37.0%, 85.4%, and 25%, respectively (all from unprocessed flour), while the highest nutritional contribution were 192.9%, 111.6%, 270.4%, and 89.9%, respectively (all from extruded flour). For women of reproductive age (15–50 years), the lowest nutritional contribution for iron, zinc, protein, and energy were 23.8%, 23.7%, 38.1%, and 26.2%, respectively, while highest nutritional contribution were 250.1%, 69.7%, 127.4%, and 62.7%, respectively, all from extruded flour. Based on these results, it showed that processed biofortified bean flour can highly improve nutritional status of consumers.

## Conclusion

Malting/roasting and extrusion processes improve nutritional and physicochemical characteristics of biofortified beans. In the present study it is was found that both processing methods increase the mineral bioavailability and improve in vitro protein digestibility of bean flour. Extruded products had higher mineral bioavailability and in vitro protein digestibility than malted/roasted samples. The pasting properties of bean flour were modified by processing, with extruded flour exhibiting lowest pasting viscosities and pasting temperature compared to malted/roasted samples, and malted/roasted samples exhibiting lower pasting viscosities than raw samples. Products from conventional bean flour (K131) and products from biofortified bean flour (ROBA1) are equally acceptable by consumers. Gruel or sauce prepared from processed biofortified beans exhibited higher nutrients and energy density than gruel/sauce prepared from unprocessed biofortified bean flour. It can be thus concluded that processed biofortified bean flour can be utilized to prepare highly acceptable nutrient and energy dense sauce or gruel suitable for feeding nutritional vulnerable populations.

## Conflict of Interest

None declared.
